# Volumetric hippocampal changes in glioblastoma: a biomarker for neuroplasticity?

**DOI:** 10.1007/s11060-023-04315-5

**Published:** 2023-05-13

**Authors:** Alessandro Zilioli, Francesco Misirocchi, Carlotta Mutti, Beatrice Pancaldi, Elisa Mannini, Marco Spallazzi, Liborio Parrino, Davide Cerasti, Maria Michiara, Irene Florindo

**Affiliations:** 1 Department of Medicine and Surgery, Unit of Neurology, University of Parma, Parma, Italy; 2 Sleep Disorders Center, Department of Medicine and Surgery, University of Parma, Parma, Italy; 3 Neuroradiology Unit, University Hospital of Parma, Parma, Italy; 4 Department of Medicine and Surgery, Unit of Oncology, University of Parma, Parma, Italy; 5 Department of Medicine and Surgery, Unit of Neurology, University Hospital of Parma, Parma, Italy

**Keywords:** Glioblastoma, Hippocampus, VBM, Neuroplasticity

## Abstract

**Purpose:**

The pleiotropic effect of gliomas on the development of cognitive disorders and structural brain changes has garnered increasing interest in recent years. While it is widely accepted that multimodal therapies for brain cancer can foster cognitive impairment, the direct effect of gliomas on critical cognitive areas before anti-tumor therapies is still controversial. In this study, we focused on the effect of IDH1 wild-type glioblastoma on the human hippocampus volume.

**Methods:**

We carried out a case-control study using voxel-based morphometry assessment, analyzed with the Computational Anatomy Toolbox software. Glioblastoma diagnosis was performed according to the latest 2021 WHO classification. Due to stringent inclusion criteria, 15 patients affected by IDH1 wild type glioblastoma were included and compared to 19 age-matched controls.

**Results:**

We observed a statistically significant increase in the absolute mean hippocampal volume (p = 0.017), as well as in the ipsilateral (compared to the lesion, p = 0.027) and the contralateral hippocampal volumes (p = 0.014) in the group of patients. When the data were normalized per total intracranial volume, we confirmed a statistically significant increase only in the contralateral hippocampal volume (p = 0.042).

**Conclusions:**

To the best of our knowledge, this is the first study to explore hippocampal volumetric changes in a cohort of adult patients affected by IDH1 wild-type glioblastoma, according to the latest WHO classification. We demonstrated an adaptive volumetric response of the hippocampus, which was more pronounced on the side contralateral to the lesion, suggesting substantial integrity and resilience of the medial temporal structures before the initiation of multimodal treatments.

## Introduction

Recent discoveries in the field of neuro-oncology have been revealing a complex relationship between brain tumors and cognitive impairment [1].

While neural circuits and areas involved in the determining of cognitive impairment have been partially outlined, the topographic correspondence between brain tumor location and cognitive deficits is not univocal. For instance, memory disorders closely resembling Alzheimer’s disease may occur in patients with high grade brain tumor located in areas typically spared in the Alzheimer’s neurodegenerative process. [2].

Studies have shown that multi-modal antineoplastic approaches, particularly radiation treatment more than chemotherapy, could damage hippocampal areas, which are crucial in memory processing [2, 3]. However, the influence of the neoplastic cells per se on mesial temporal structures is far from a definite understanding.

Gliomas are the most common intracranial brain tumors [4], and they represent a highly heterogeneous group associated with potentially different effects, depending on the specific tumor.

It has been suggested that gliomas overall provoke an intense struggle between neuroplasticity and destructive processes in the brain, with effects extended beyond the tumor’s primary location. The detrimental effects are thought to be caused by pro-inflammatory extracellular micro-environment [5] and elevated glutamate levels [6], ultimately leading to widespread neuronal death. On the other hand, the hippocampus is known to have intrinsic resilience to tumor cell invasion and its effects [7], as well as the ability to remodel itself in response to various stimuli, thanks to the persistence of neural stem cells [8].

Therefore, the final effect of glioma on the hippocampus depends on the balance between the tumor-related toxic effects and the resilience of this brain structure.

Given the lack of anatomopathological studies focusing on tissue changes within the hippocampus in the early stages of tumor growth, the assessment of volumetric value of this area may represent an indirect approach to define its integrity [9]. In this perspective, post-processing imaging techniques may assume a pivotal importance, particularly if combined with the recent WHO classification, which allows for more specific categorization of brain tumors in terms of molecular features.

In this study, we analyzed the hippocampal volume in a highly selected group of adult patients newly diagnosed with isocitrate dehydrogenases 1 (IDH1) wild-type glioblastoma, compared to age-matched healthy controls, in order to test hippocampus resilience in brain affected by tumors.

## Materials and methods

### Patient selection and study design

Data were retrospectively analyzed on 131 adult patients affected by glioblastoma, diagnosed from January 2013 to December 2022 at University Hospital of Parma, Italy.

**Fig. 1:**
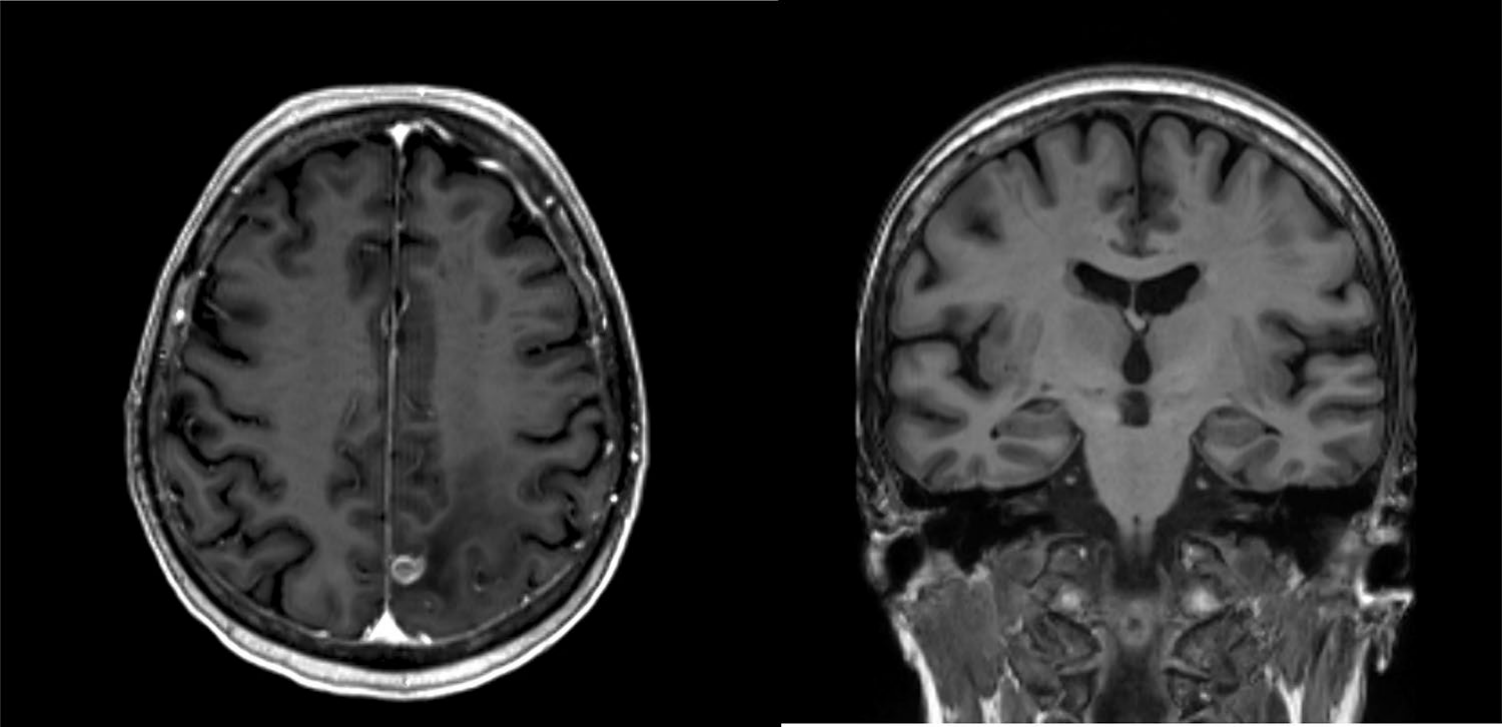
On the left, the example of a glioblastoma with minimal local mass effect and perilesional edema. On the right, the presence of hippocampal-sparing, which constituted a strict inclusion criterium in order to ensure reliable pre-processing analysis

The inclusion criteria were the following:

1. Age range between 50 and 80 years (patients exceeding this threshold were excluded for a more precise epidemiological matching with the control group).
2. Availability of a high-resolution (T13D) brain Magnetic Resonance Imaging (MRI) representing the first diagnosis of glioblastoma. Cases displaying involvement or significant mass effect on mesial temporal structures, supported by FLAIR and gadolinium-enhanced T1 sequences, were excluded (see Fig. 1).
3. Histopathological confirmation of glioblastoma IDH-1 wild-type according 2021 WHO Classification of Tumors of the Central Nervous System [10], as well as molecular and immunophenotypic characterization.
4. Absence of other significant neurological comorbidities.
5. Absence of hippocampal pathology at first MRI visual inspection (e.g. focal cortical dysplasia, hippocampal sclerosis, incomplete hippocampal inversion).

Due to the strict inclusion criteria, the final sample was composed of 15 patients over the total of 131. Brain MRI from 19 age-matched healthy subjects were used as controls.

The first aim of this study was to estimate hippocampal volumetric differences between patients and controls, at the time of initial GBM diagnosis. The study considered mean, ipsilateral and contralateral (in relation to the site of the lesion) hippocampus, both as absolute and as normalized volume.

We investigated if the volumetric changes included a reference area, in order to ascertain whether the volumetric alterations triggered by GBM were localized or distributed across various cerebral regions. Since patients with GBM presented the involvement of heterogenous brain regions, mostly located in the supratentorial areas, we decided to examine the volume of the cerebellum, as representative of non-affected tissue. In addition, the primary involvement of GBM in this brain area is extremely rare [11].

Furthermore, we assessed brain tumor volume with gadolinium-enhanced T1 and FLAIR sequences using the orthogonal ellipsoid method [12], as previous studies demonstrated the presence of neoplastic cells even beyond areas of gadolinium enhancement [13].

All subjects were enrolled after obtaining written informed consent, and the study was carried out in accordance with Helsinki principles and with approval from the local ethical committee.

### MRI acquisition

The entire population of patients and controls enrolled in our study underwent brain MRI scans using a 3 Tesla scanner (GE Healthcare Discovery MR 750) equipped with an 8-channel head coil, at the Neuroradiology Department of University of Parma.

The imaging protocol, designed for brain tumor assessment, included: pre-post contrast 3D T1 weighted (BRAVO, section thickness 0.9 mm, TR/TE 12.36/5.18 ms, flip angle 13°), fluid attenuated inversion recovery (FLAIR), gradient-echo (GRE) and diffusion weighted imaging (DWI). The MRI protocol was the same, except for the contrast medium, which was excluded for the control group.

### Data postprocessing

We performed a voxel-based morphometry (VBM) analysis on anatomical T1-3D images using the CAT12 toolbox (r1355, Structural Brain Mapping group, Jena University Hospital, Jena, Germany, http://www.neuro.uni-jena.de/cat/), running in SPM12 (Statistical Parametric Mapping; Wellcome Trust Centre for Neuroimaging, London, UK) on MATLAB.

To ensure accurate analysis, we manually reoriented the high-resolution images to the anterior commissure (mm coordinate: 0, 0, 0), which represents the origin. Using the “segment data” module of CAT12, we obtained the gray matter (GM), white matter (WM) and cerebrospinal fluid (CSF) from the corresponding anatomical images, and extracted region of interest (ROI) values using the neuromorphometric atlas; we also obtained the total intracranial volume (TIV) for each subject.

To ensure homogeneity of the sample and verify segmentation results, we examined potential outliers, visually inspected all processed images, and confirmed that the pre-processing quality was satisfactory with a B value for the summary report provided by CAT12. Next, we modulated the GM images and smoothed them with an 8-mm full width at half-maximum Gaussian kernel.

For each subject we measured bilateral hippocampus volumetry expressed both as absolute data and as normalized volume (hippocampus volume/TIV).

### Statistical analysis

All quantitative data were expressed as mean and standard deviation (SD). On SPM we adopted a two-sample t-test to analyze the difference in GM volumes between patient and control groups with sex, TIV, and education as covariates. We set an absolute masking threshold of 0.2 and a p value < 0.05, with a spatial extent threshold of 50 voxels as significant. To investigate the structural changes in the hippocampus, the WFU Pickatlas toolbox was used to create masks of the bilateral hippocampal region of interest (ROI), based on the Anatomical Automatic Labeling (AAL) template.

Additionally, the ROI-based hippocampal volumes were extracted using the “extract ROI data” module on CAT12 and the data were analyzed with the open-source statistical software Jamovi v. 2.3.21.0.

We analyzed the following brain volumes (absolute and normalized value):

- Mean value of hippocampus in patients and controls.
- Value of the ipsilateral hippocampus (compared to the site of the lesion) relative to the mean values of controls’ hippocampi.
- Value of the contralateral hippocampus (compared to the site of the lesion) relative to the mean values of controls’ hippocampi.

Finally, we decided to assess volumetric changes encompassing the cerebellum, a brain region spared by the tumoral growth in our sample.

A descriptive statistic was used to summarize the population characteristics.

## Results

We enrolled a total of 15 patients (8 females) with glioblastoma and 19 controls (14 females) (see Table 1 for detailed demographic data).

**Table 1:** Summary of population characteristics and anatomopathological tumor data

	Patients	Controls
Numbers	15	19
Sex ratio, M/F	7/8	5/14
Age at diagnosis (SD)	64.3 (± 8.08)	63.5 (± 6.90)
Level of education (years, SD)	11.3 (± 4.50)	13.5 (± 4.36)
Seizures	9	NA
Lesion site (n)	6 frontal, 3 temporal, 1 parietal, 5 multifocal	NA
IDH wild-type glioblastoma	15	NA
Codeletion 1p/19q	0	NA
TIV (cm^3^, SD)	1504 (± 101)	1491 (± 202)
Tumor Volume (T1 gadolinium, cm^3^)	9.40 (± 9.45)	NA
Tumor Volume (FLAIR, cm^3^)	28.4 (± 27.7)	NA

Histological confirmation of glioblastoma was obtained for all cases, either by surgical removal (13 patients) or biopsy (2 patients).

The two groups were comparable in terms of age (one-way ANOVA, p = 0.745), education (one-way ANOVA, p = 0.160), and sex (χ^2^ test, p = 0.218).

Moreover, no significant difference was found between the TIV of patients (mean value 1504 cm^3^, ± 101) compared to controls (mean value 1491 cm^3^, +- 202, p = 0.820).

Brain tumors were localized in the frontal lobe (6 patients), extra-hippocampal temporal lobe (3 patients), and parietal lobe (1 patient), multifocal in 5 patients.

Mean hippocampus volume was 3.35 cm^3^ (± 0.286) and 3.12 cm^3^ (± 0.294) in patient and control group, respectively.

The hippocampus on the same side as the lesion (ipsilateral) had an absolute mean volume of 3.31 cm^3^ (± 0.250) and a normalized mean value of 0.221 (± 0.020). The contralateral hippocampus had an absolute mean volume of 3.38 cm^3^ (± 0.335) and a normalized mean value of 0.225 (± 0.021).

The patient group had a statistically significant increase in the absolute mean hippocampal volume (p = 0.017) and in the absolute ipsilateral hippocampal volume (p = 0.027), which did not survive the normalization per TIV (respectively p = 0.061 and p = 0.106).

The patient group had also a statistically significant increase in both the absolute (p = 0.014) and normalized (p = 0.042) contralateral hippocampal volume.

The mean tumor volume was 9.40 cm^3^ (± 9.45) and 28.4 cm^3^ (± 27.7) for T1 gadolinium and FLAIR sequences, respectively.

The assessment of volumetric changes in the cerebellum showed a statistically significant reduction (p < 0.01) in ipsilateral and contralateral hemispheres in the group of patients, with regard to both absolute and normalized values.

**Fig. 2:**
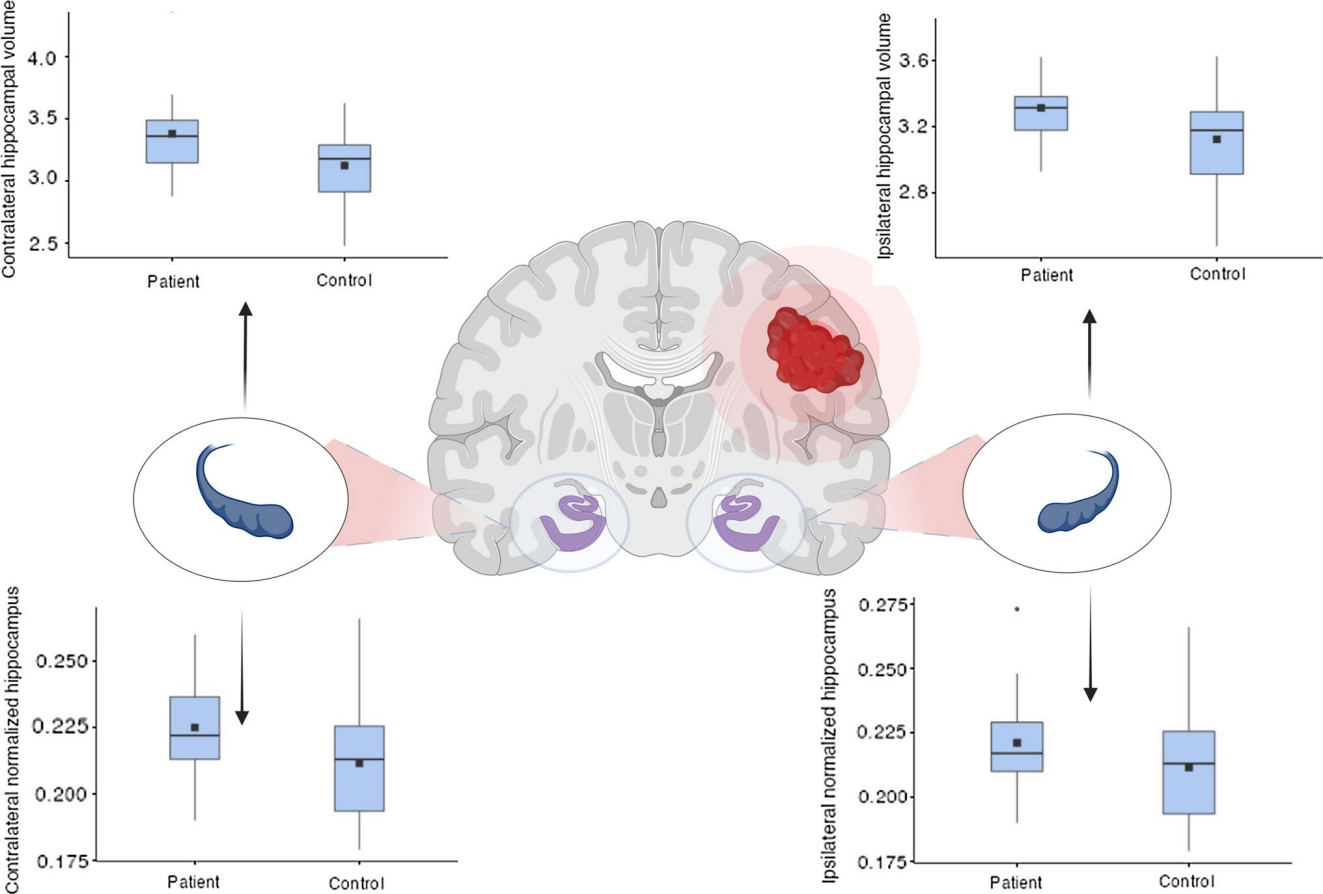
The effect of glioma on the hippocampal volume. Box plots showing both the mean absolute and normalized ipsilateral and contralateral hippocampal volume of patients compared to the mean hippocampal value of controls. The square indicates the mean value of the group, the horizontal line indicates the median. The mean absolute values are expressed in cm^3^, while the normalized values are calculated as the ratio of absolute value to TIV. Created with BioRender.com.

Statistical results are summarized in Table 2 and Fig. 2.

**Table 2:** Hippocampal volume comparison between patients and controls, considering mean, ipsilateral and contralateral (based on lesion site) values

	Patients	Controls	P Value
Mean Hippocampal Volume (HV) ( SD)			
HV	3,35 cm^3^ (± 0.286)	3.12 cm^3^ (± 0.294)	**p = 0.017**
Normalized per TIV	0.233 (± 0.019)	0.212 (± 0.022)	p = 0.061
Ipsilateral Hippocampal Volume (HV) ( SD)			
HV	3.31 cm^3^ (± 0.250)	3.12 cm^3^ (± 0.294)	**p = 0.027**
Normalized per TIV	0.221 (± 0.020)	0.212 (± 0.022)	p = 0.106
Contralateral Hippocampal Volume (HV) (SD)			
HV	3.38 cm^3^ (± 0.355)	3.12 cm^3^ (± 0.294	**p = 0.014**
Normalized per TIV	0.225 (± 0.021)	0.212 (± 0.022)	**p = 0.042**

Volumes are reported as absolute values and after normalization for TIV (total intracranial volume). On the right, a t-test analysis considering the hypothesis of a larger hippocampal volume in patients affected by glioblastoma

## Discussion

Our study explored the relationship between hippocampal volume and IDH wild-type glioblastoma at the time of diagnosis. We found an overall increase of the hippocampal volume, more significant in the contralateral side with respect to the neoplastic lesion.

In addition, we detected an unexpected volumetric decrease in the cerebellar hemispheres, which was chosen as control brain area, due to its rare involvement in the disease evolution.

The potential effects of brain tumors on mesial temporal structures have been suggested by few reports, leading to conflicting results [9, 14, 15].

Research in the field has to deal with intrinsic complexity and heterogeneity, taking into account the different molecular subtypes of gliomas (e.g., IDH wild type vs. mutation), grading (low- and high grade), classification in use, and typologies of therapies. To disentangle such considerable variability, we used strict inclusion criteria, we adopted the novel WHO diagnostic criteria for gliomas, selecting a unified cohort of IDH1 wild-type glioblastoma and we performed volumetric assessment before any surgical or radio-pharmacological treatment.

Our results are in line with Yuan et al. [15] who analyzed a more heterogeneous cohort of either low- or high-grade gliomas, showing that both ipsilateral and contralateral hippocampi were significantly increased in terms of volumetric response and dynamic regional neuronal activity. The unexpected correlation between a more adaptive response in the mesial temporal areas and a shortened survival was possibly explained by the fact that a more aggressive lesion with rapid proliferation and tissue damage yields to a stronger compensatory reaction in the hippocampal areas, even if not enough to counterbalance the disease aggressiveness.

On the other hand, Karunamuni et al. [9] reported a reduction in hippocampal volume in patients with glioma prior to radiotherapy, compared to a group of controls. However, this finding might be related either to the difference in the time of the neuro-radiological acquisition (after the surgical removal of the lesion) or to the inner heterogeneity of enrolled patients, affected by various subtype of tumors, such as GBM, oligodendroglioma and oligoastrocytoma.

It is well-known that hippocampus is highly sensitive to a large number of stimuli, such as ischaemia, trauma and physical exercise, which can all induce variations in its morphology and function [16].

We theorize that the presence of the GBM, with both its local and widespread detrimental effects, may induce a compensatory response within the hippocampus, one of the few sites having a neurogenesis capability in the adult brain [17]. Brain tumors may thus represent an additional provocative factor for hippocampal plasticity. The higher proximity of the brain tumor may justify the volumetric differences observed between ipsi and contralateral mesial temporal areas, with ipsilateral limbic areas being more affected by the local cytotoxic effect exerted by the tumor [6] disrupting local brain networks.

These results provide further evidence supporting a significant interaction between the hippocampus and glioblastoma. Intriguingly, previous research has suggested that the hippocampus may have a dual role in this relationship. It contains neural stem cells that can potentially contribute to the development of neoplastic cells through aberrant replication, and conversely, the mesial temporal areas are known to create an inhospitable microenvironment that impedes extra-temporal tumor dissemination [18].

Moreover, the finding of substantial integrity and resilience in the mesial temporal areas before to the start of the multimodal therapies, suggests that the prominent memory deficits emerging during the course of the disease may be related to the radio- chemotherapy treatments, rather than being a direct consequence of the glioma. The implementation of targeted radiation treatments and reduced chemotherapy-induced hippocampal toxicity, accompanied by cognitive rehabilitation programs, may be highly effective in preventing the development of hippocampal-related cognitive impairments.

The discovery of reduced volume within the cerebellar hemispheres provides compelling evidence that neuroplasticity is not a property that is uniformly distributed throughout the brain’s neurons, but rather, it may be a localized process. This conclusion is reinforced by the fact that the TIVs were similar across the groups.

We postulate that the presence of a glioma may lead to crossed-diaschisis in the cerebellum, resulting in volumetric reduction. This finding has previously been associated with worse clinical outcomes, impaired supratentorial cerebrovascular reactivity, as well as marked hypometabolism in FDG-PET [19].

The main limitation of our study lies in the small sample size. Nevertheless, we were able to identify statistically significative findings about the volumetric increase of the contralateral hippocampus in the group of patients. Reasonably a larger sample may confirm our results and could reinforce the evidence for a trend of an increase volume even in the ipsilateral one. Moreover, the loss of statistical significance data of ipsilateral hippocampus when normalizing for TIV indicates a susceptibility of the data itself to bias induced by brain volumes and therefore requires validation in further studies.

We acknowledge that our cohort size was limited due to the rigorous patient selection criteria (see Fig. 1.) but we prioritized conducting a dependable pre-processing analysis with greater reproducibility. Unfortunately, the absence of a histological analysis of the hippocampal tissues precludes our ability to comprehend the underlying tissue alterations that mark neuroplasticity.

## Conclusions

To our knowledge, this is the first study to investigate volumetric hippocampal changes in a cohort of IDH1 wild-type glioblastoma patients according to the latest WHO classification. Our findings reveal a potentially intriguing hippocampal compensatory neuroplasticity, which is an emerging concept in the field of neuro-oncology. We observed an adaptive response of the hippocampus, with an increased volumetric value more pronounced on the side contralateral to the lesion, in a cohort of adult IDH1 wild-type glioblastoma patients.

These results suggest the substantial integrity of the mesial temporal structures and their potential resilience in the presence of an extra-hippocampal glioma, opening the way to the relevance of a cognitive rehabilitation program during the course of the disease.

## Data Availability

The datasets generated during and/or analysed during the current study are available from the corresponding author on reasonable request.
